# Recent Advances in Kidney Bioengineering

**DOI:** 10.3389/fped.2021.743301

**Published:** 2021-11-25

**Authors:** Nina Cintron Pregosin, Robert Bronstein, Sandeep K. Mallipattu

**Affiliations:** ^1^Division of Nephrology, Department of Medicine, Stony Brook University, Stony Brook, NY, United States; ^2^Graduate Program in Molecular and Cellular Pharmacology, Stony Brook University, Stony Brook, NY, United States; ^3^Renal Section, Northport VA Medical Center, Northport, NY, United States

**Keywords:** organoids, 3D bioprinting, bioengineering, decellularization, microfluidics, organ-on-a chip

## Abstract

Kidney disease is an epidemic that affects more than 600 million people worldwide. The socioeconomic impacts of the disease disproportionately affect Hispanic and non-Hispanic Black Americans, making the disease an issue of social inequality. The urgency of this situation has only become worse during the COVID-19 pandemic, as those who are hospitalized for COVID-19 have an increased risk of kidney failure. For researchers, the kidney is a complex organ that is difficult to accurately model and understand. Traditional cell culture models are not adequate for studying the functional intricacies of the kidney, but recent experiments have offered improvements for understanding these systems. Recent progress includes organoid modeling, 3D bioprinting, decellularization, and microfluidics. Here, we offer a review of the most recent advances in kidney bioengineering.

## Introduction

Kidney disease is a public health crisis that affects 37 million people in the United States. As many as 9 in 10 adults with chronic kidney disease (CKD) may not even know they have the condition. Progression of the disease can lead to kidney failure and end-stage renal disease (ESRD), which requires dialysis or a kidney transplant (1). There are currently more than 100,000 people on the organ transplant list waiting to receive a kidney, and the average wait time is between 3 and 5 years (2). Despite the life-saving measures of dialysis, after 5 years on dialysis the survival rate drops to just 33% (3). While the number of kidney transplants has steadily increased since 2015, the number of patients waiting for a transplant has remained the same. In addition, as many as two-thirds of kidney transplants come from deceased donors, yet ~20% of kidneys recovered from these donors are discarded due to abnormal histology or biopsy findings (2). Furthermore, the number of kidneys being discarded increased 91.5% from 2000 to 2015, without a change in the quality of these organs (3).

Conditions affecting the kidney incur an enormous cost given the time-course of pathological progression. In 2018, CKD cost Medicare $81.8 billion, representing 23% of Medicare fee-for service spending (4), with ESRD, the end result of CKD, costing an additional $34 billion. The high cost and lack of access to treatment, including dialysis, is another issue which disproportionately affects Hispanic and non-Hispanic Black Americans, as 14% of Hispanic adults and 16% of non-Hispanic Black adults suffer from CKD (1). While Black Americans represent ~13% of the population in the United States, they make up more than 30% of patients with ESRD. In addition, Black Americans are less likely to be referred for transplant evaluation. Despite increasing rates of transplantation, Black Americans also have worse graft survival than Caucasian Americans (5).

The gravity of this crisis has been exacerbated during the COVID-19 pandemic, with ~30% of hospitalized patients with COVID-19 experiencing some form of kidney dysfunction. According to a recent study from Columbia University, up to 40% of COVID-19 patients in the intensive care unit will experience kidney failure (6). Another study from Stony Brook University reported that both proteinuria and hematuria at the time of admission were associated with poor outcomes in hospitalized patients with COVID-19 (7). In addition, in patients requiring mechanical ventilation, acute kidney injury was the highest predictor of mortality (8).

The White House and Department of Health and Human Services addressed the epidemic of kidney disease by announcing the *Advancing American Kidney Health* initiative in 2019. The initiative encourages the development of artificial kidneys and supports research toward improving diagnostics and biomarkers for kidney disease. One of the major goals of this initiative is to reduce the number of Americans with ESRD by 25% and double the number of available kidneys for transplant by the year 2030 (9). As such, advancements in the field of kidney bioengineering are essential to meet these goals. In this review, a small number of recent advancements in various facets of kidney bioengineering are described.

## Kidney Organoids

To identify novel strategies for the treatment of kidney disease, we must create better models to study its molecular and cellular etiology. This can be accomplished by engineering “miniature kidneys” in the form of kidney organoids. Organoids are three-dimensional, self-patterning cell cultures that can model the basic structure and function of organs. In comparison to traditional two-dimensional cell cultures, organoids better represent the *in vivo* microenvironment of the native kidney, making them useful models for studying the pathogenesis of kidney disease (10). Kidney organoids have already been utilized to study kidney tubular damage and model genetic causes of kidney disease. Specifically, organoids were found to be more effective for modeling polycystic kidney disease than the standard two-dimensional cell cultures (11). Furthermore, dozens of organoids can be generated in a single well of a 96-well plate, thereby making them useful for high-throughput screening of novel small molecules for the treatment of kidney disease as well as to test nephrotoxicity of commonly used agents in the clinic (12).

**Figure 1:**
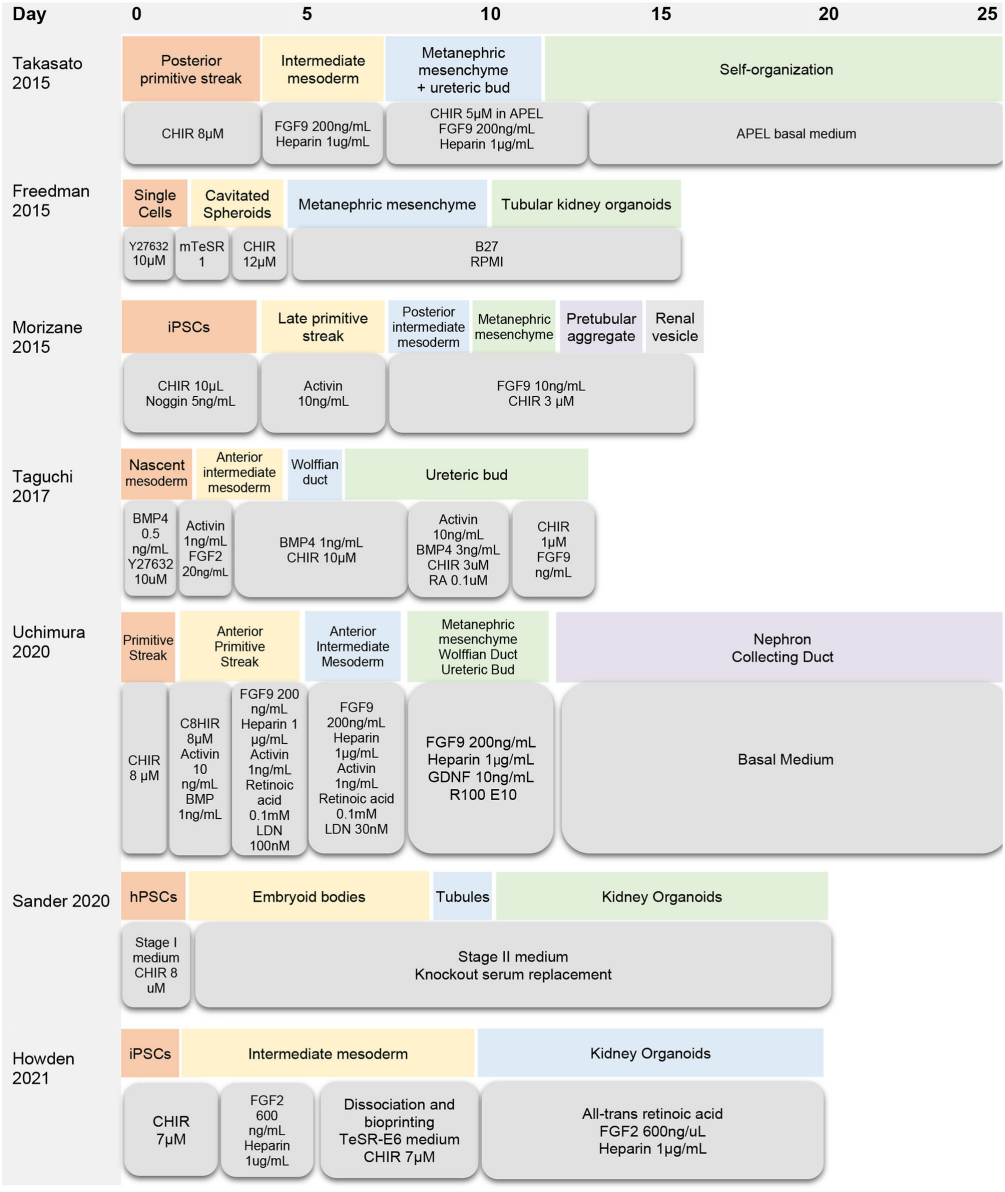
Timeline of organoid differentiation protocols.

There are several protocols for generating kidney organoids from embryonic stem cells and induced pluripotent stem cells (Figure 1, Table 1). Van Den Berg and colleagues demonstrated that organoids implanted underneath the kidney capsule of mice were capable of growth and expansion—without the addition of exogenous growth factors (20). The implanted organoids displayed early signs of vascularization and glomerular maturation, in comparison to organoids cultured *in vitro*, which remained disorganized and immature (20). Modulating Wnt, a family of glycoproteins that regulate embryogenesis and stem cell renewal, has also been shown to influence organoid development (21, 22). Low et al. also demonstrated that modulation of Wnt signaling induced the formation of vascularized organoids that continued to mature after implantation in mice. In addition, increased Wnt signaling contributed to the formation of larger kidney organoids (23).

**Table 1:** Advantages and disadvantages of kidney organoid differentiation protocols.

**References**	**Starting cell type**	**Organoid composition**	**Disadvantages**
Takasato et al. (13)	hPSCs MEF feeder layer	Podocytes Proximal and distal tubule cells Collecting duct cells	Capillary loops do not form in glomeruli
Freedman et al. (14)	hPSCs	Nephron progenitors Podocyte-like cells Proximal and distal tubule cells	Lacks vascular formation Tubules do not contain full brush border No evidence of ureteric bud
Morizane et al. (15)	hESCs and iPSCs	Nephron progenitors Podocyte-like cells Renal vesicle-like cells Proximal and distal tubule-like cells	Lacks ureteric bud progenitors Lacks vascular progenitors
Taguchi et al. (16)	iPSCs	Ureteric bud-derived collecting duct-like cells Nephron progenitors	Unable to induce the formation of stromal progenitors
Uchimura et al. (17)	iPSCs	Collecting duct cells Ureteric bud cells Principal cells Intercalated cells Urothelium cells Proximal tubule cells	Low yield of endothelial cells
Sander et al. (18)	hPSCs	Podocytes Proximal and distal tubular cells Connecting duct epithelia	Extended culture yields non-renal cell types
Howden et al. (19)	iPSCs	Podocytes Nephron progenitors Endothelial cells Distal tubule-like cells Stromal clusters Ureteric epithelial cells	Requires induction of ureteric epithelium from distal nephron- containing organoid Protocol requires additional 2– 3 weeks

### Limitations of Kidney Organoids

Despite the many applications of kidney organoids, there are significant questions that must be answered before these “miniature organs” can be used as a form of renal replacement therapy. One major limitation of using kidney organoids for research is that they lack complexity and nephron maturity (14, 15). Even the most advanced organoids have immature vasculature, resembling a first-trimester fetal kidney (13). In addition, prolonging the growth of kidney organoids has not been shown to improve their differentiation. Size is another limiting factor because kidney organoids grown on the millimeter scale often develop a hypoxic core and metabolic deficiency (20). Current methods of organoid differentiation can also be difficult to reproduce due to batch-to-batch variation and uncontrolled patterning of the differentiating tissue (24). Off-target cells are also a concern, since it is estimated that as much as 20% of the cellular population within each organoid is of non-kidney origin (25).

## Biological and Non-biological Scaffolds

Biological and non-biological scaffolds may improve the complexity of kidney organoids by providing a structure that supports their maturation. Biological scaffolds usually include growth factors that promote cell proliferation (Table 2), while non-biological scaffolds offer structural support to the cells (Table 3). These scaffolds can influence organoid differentiation based on their stiffness (41). In addition, dissolvable scaffolds can offer support while the cells are being seeded and then degrade once the cells are distributed into the pattern of interest. These dissolvable scaffolds are particularly useful for creating three-dimensional shapes with hollow channels that represent vascular-like structures. Notably, Homan et al. used this technique to model the 3D structure of the renal proximal tubule. A silicone gasket was used as a supportive structure to house an ECM mixture of gelatin and fibrinogen. A convoluted tubule-like structure made of a dissolvable polymer was then laid on top of the ECM mixture and embedded in an additional layer of ECM. After the ECM cured, the dissolvable polymer was evacuated to leave a hollow structure in which epithelial cells were perfused (42). This three-dimensional model of the proximal tubule was created using 3D bioprinting.

**Table 2:** Properties of common biological scaffolds.

**Scaffold**	**Composition**	**Advantages**	**Disadvantages**	**References**
Agarose	Agarobiose backbone chain of D-galactose	Biodegradable Structural stability Cell viability Rapid gelation	Low cell adhesion Cell spreading Limited ability to support cell growth	(26, 27)
Alginate	β-D-mannuronic acid and α-L- guluronic acid	Inexpensive Cross-linkable	Highly hydrophilic Minimal protein absorption	(27)
Collagen	Type I collagen	Most commonly used scaffold Low immunogenicity Cross-linkable	Low mechanical properties Low stability	(28)
Dextran	α-1,6-linked D- glucopyranose residues	Biodegradable (dextranase)	Poor mechanical strength	(29)
Fibrin	Fibrinogen	Regenerative capacity High viscosity Cross-linkable	Low shape fidelity	(30)
Gelatin	Denatured collagen	Low cost Simple processing Low antigenicity Cross-linkable	Low mechanical properties Low stability	(31, 32)
Hyaluronic acid	D-glucuronic acid and N-acetyl-d-glucosamine	Formation of flexible hydrogels UV cross-linkable	Slow gelation rate Poor mechanical properties	(27)
Matrigel	ECM proteins (collagen, laminin, entactin, etc.) derived from Engelbreth-Holm- Swarm mouse sarcoma cells	Cross-linkable Peptides and growth Factors assist cell growth	Poor mechanical strength Poor printability Temperature-sensitive	(29, 31)

**Table 3:** Properties of common non-biological scaffolds.

**Scaffold**	**Composition**	**Advantages**	**Disadvantages**	**References**
Carbohydrate glass	Sucrose, glucose	Dissolvable Cytocompatible	Brittle at room temperature	(33)
GelMA	Gelatin methacrylate	UV cross-linkable High mechanical strength Stable in media months post-printing	Low viscosity and printing resolution at low concentrations	(34, 35)
PCL	Polycaprolactone	High mechanical strength Low toxicity Slow degradation rate	Requires printing at high temperatures Not useful for live cell printing	(36, 37)
PDMS	Polydimethylsilo-xa ne	Widely used for organ- on-a-chip devices Low cost	Hydrophobic Poor cell attachment	(38, 39)
**High structural integrity Biocompatible**				
PEG	Polyethylene glycol diacrylate and polyethylene glycol-methacrylate	Biocompatible UV cross-linkable	Poor mechanical strength Poor cell and protein adhesion	(27, 37)
PLA	Polylactic acid	Widely used for 3D printing Low-cost Biodegradable	Poor thermal stability Printed at high temperatures Not useful for live cell printing	(37, 40)
Pluronic	Poloxamers	Widely used for 3D bioprinting UV cross-linkable Biodegradable	Unstable Erodes within hours	(37)
Silicone	Polysiloxane	Biocompatible Mechanically durable UV cross-linkable Low toxicity	Relatively expensive Poor cell interaction	(32)

### 3D Bioprinting

3D bioprinting is a useful technique for engineering both scaffolds and tissues. This technology is similar to 3D printing, but rather than using plastic filaments, bioprinters use cells suspended in “bioink” to create sophisticated three-dimensional structures with precise geometry and scaffold porosity (43). In the example from Homan et al., the dissolvable tubule-like structure was designed and printed using a sacrificial Pluronic bioink that, when cooled to 4°C, liquefied to leave a hollow channel in its place (42). Ideally, bioinks are hydrogels that have adequate mechanical, chemical, and biological characteristics for supporting cell growth. These inks may be composed of substances that resemble the microenvironment of a particular cell line, such as collagen, alginate, or gelatin, or they can be made of synthetic materials (Table 3). One advantage of 3D bioprinting is that bioinks such as Derma-matrix and Novogel are commercially available (31). However, the choice of appropriate bioink is often dependent on the type of 3D bioprinter.

### Types of 3D Bioprinters

Extrusion-based printing is the most used method for bioprinting cells. Extrusion-bioprinting uses mechanical force to push hydrogels through a pressurized syringe and generate 3D cellular structures (44). This technique is useful for printing viscous bioinks and can print structures as small as 200 μm in diameter. Recently, extrusion-bioprinting was used to successfully print iPSCs for the generation of kidney organoids (12). Due to the shear stress produced during the printing process, overall cell viability for extrusion-based bioprinting is between 40 and 80% (43, 44).

Inkjet-based bioprinting is most like printing ink on paper, as droplets of bioink are quickly released onto a particular substrate. Graham et al. utilized this technique to print both HEKs and mesenchymal cells in aqueous droplets 1 nL in volume. The researchers generated structures <200 μm in diameter that had an average cell viability of 90% post-printing. In addition, the bioprinted cells continued to proliferate several weeks post-printing (45). However, inkjet-based bioprinting is generally limited to using liquid bioinks and can still cause thermal and mechanical stress to cells. A newer strategy, laser-assisted bioprinting, uses a laser to deposit materials onto a metal film and a receiving substrate. This technique has a high resolution between 10 and 50 μm and cell viability is more than 95%; however, this process is expensive and prone to metallic contamination due to the use of the metal film (46). Stereolithography is an alternative approach to laser bioprinting that uses illumination to solidify printed polymers such as acrylics and epoxies. Stereolithography has a resolution of 1.2–200 μm, a cell viability of about 90%, and has been utilized to print iPSCs. Despite its impressive resolution, stereolithography is limited by the type of bioink used, as this technology can only be properly employed to print curable photopolymers (47). Therefore, toxic photo-curing agents and UV exposure may harm the cells being printed (43).

### Applications of 3D Bioprinting

Several protocols for printing simple tissue, including skin and cartilage, have already been established (48, 49). Recently, bone-marrow derived stem cells were successfully implanted in mice and continued to grow within the animal. The bioprinted structures also maintained their structural integrity post-implantation (50). In a more complex example, 3D bioprinting was used to successfully print cardiac tissue. The tissue was not only viable, but also exhibited partial functionality. In response to an electrical stimulus, the bioprinted heart tissue contracted, representing the mechanical beating of the heart (51). While there has been some progress to date on bioprinting simpler tissue structures, establishing a method for 3D bioprinting viable and functional complex organs like the kidney has been difficult since the kidney contains more than 20 unique cell types (49).

3D bioprinting is particularly useful for distributing induced pluripotent stem cells (iPSCs) in high-resolution, 96-well plate configurations. Lawlor et al. demonstrated that 3D bioprinted iPSCs generated organoids that displayed a similar morphology to manually pipetted organoids—all while using a lower initial number of cells. RNA sequencing data revealed increased expression of podocyte and renal progenitor genes in the bioprinted structures, with no significant difference in the number of cells per population across each sample. This study also showed that the end products of organoid bioprinting can be more easily replicated, because bioprinted structures exhibited uniformity across multiple wells within the 96-well plate. This bioprinting method is amenable to high throughput analysis and was validated by using the organoids as substrates for drug screening and nephrotoxicity testing (12). 3D bioprinting also allowed the researchers to uncover the effects of cellular distribution on organoid development. For example, iPSCs printed in a straight line, rather than the standard dot configuration, showed signs of increased membrane transport, extracellular organization, and cell adhesion. This elongated shape also promoted a more uniform distribution of nephrons throughout the organoid. This eliminated the formation of a hypoxic core that often compromises the growth and survival of organoids (12).

### Limitations of 3D Bioprinting

Despite advances in 3D bioprinting technology, there are still numerous limitations. In theory, 3D bioprinters are capable of printing complex tissues. However, the major limiting factor is the ability of cells to survive the printing process. Print speed, temperature, and pressure are all factors to take into consideration when working with different cell types. Even after kidney organoids are successfully bioprinted, the most complex three-dimensional tissues can only maintain their shape for a maximum of 6 months (43). Continued optimization in bioink formulation, extrusion time, pressure, and UV crosslinking are necessary to ensure long-term cell survival post-bioprinting.

### Perfusion Decellularization

As a result of the challenges associated with creating and printing biological scaffolds, methods for deriving the naturally occurring ECM have gained popularity. Due to their ability to alter cell proliferation, migration, and differentiation, ECM-derived scaffolds provide an optimal microenvironment for growing three-dimensional tissues (52). Most ECM-derived scaffolds are obtained by removing cells from animal tissue, leaving only structural and regulatory proteins intact. In one study, iPSCs seeded onto a decellularized matrix successfully attached and formed vessel-like structures, showing the effectiveness of these matrices as scaffolds for cell culture (53). Since the cellular content has been removed, these tissues are also considered non-immunogenic, and therefore have a lower likelihood of being rejected after implantation (54). As such, decellularized tissue is useful for medical applications, where the ECM is implanted to aid in the growth of new tissues. For instance, in breast reconstruction post-mastectomy, decellularized tissue can be placed between the muscle and breast implant. Studies have demonstrated that patients who received the decellularized tissue matrix-implant had faster healing times and few adverse effects (55).

Due to the maintenance of growth factors and structural components, decellularized tissue can also be used to create hydrogels for bioink in 3D bioprinting. For instance, Ali et al. created a bioink from decellularized whole porcine kidneys and, when used to print human kidney cells, enhanced cell viability and proliferation and showed structural characteristics of native renal tissue. This ECM-derived bioink was formulated from gelatin, HA, and glycerol to recreate the kidney-specific microenvironment (56).

### Methods for Tissue Decellularization

Chemical, physical, and biological techniques are all effective for removing cells while maintaining ECM components, growth factors, and even vascular structures (41, 57). Caralt et al. demonstrated that perfusion of 1% Triton X-100 and 0.1% SDS through the renal artery of rat kidneys was effective for clearing cells while maintaining renal microarchitecture, matrix bound fibroblast growth factor, and vascular endothelial growth factor (58). However, varying concentrations of SDS are effective yet cytotoxic, and therefore require extensive washing. SDS can also alter the microstructure of the ECM including collagen fibers. Mechanical methods for decellularization avoid such toxicity by using high temperature and pressure to lyse cells. Freeze-thawing, high hydrostatic pressure, or supercritical carbon dioxide are also reasonable options for removing cells and genetic material from tissue (41).

While decellularization offers an alternative method for kidney manufacturing, the procedure still has significant room for improvement. One major challenge is a limited supply of donor tissue for whole-organ decellularization. Some also argue that ECM-derived scaffolds lack the mechanical strength necessary to support long-term studies. In addition, while ECM components such as collagen, fibrinogen, and glycosaminoglycans are considered less immunogenic, these biomaterials can trigger an immune response by inducing the secretion of (IL)-1B and IL-6. However, the immune response can be suppressed by coating acellular materials with immune-neutral substances (59). To date, clinical applications utilizing decellularized tissue have been limited to less complex tissues, such as skin and muscle, and biological scaffolds derived from human kidneys require further investigation (41, 60).

### Kidney-on-Chip

Although biological scaffolds are useful for mimicking the microenvironment of a variety of cell types, they lack one critical component for modeling the kidney: fluid flow. Because blood is constantly flowing through the kidney, static two-dimensional tissue cultures may not accurately represent the behavior of the cells in their dynamic environment. Microfluidic chips offer a solution to this issue by combining three-dimensional cell culture with fluid flow to replicate the physiology of multiple organ types. This technology is referred to as an “organ-on-a-chip” (OOAC) and has already been well-established for creating artificial cell environments for lung, liver, and gut tissue (61). Recently, a lung-on-chip model with cells seeded on a flexible membrane was able to effectively mimic the motion of breathing (62). Microfluidic chips are also extremely reproducible, therefore avoiding the variability that is often attributed to traditional cell culture systems (63). However, it is more difficult to establish such a system for the kidney due to its complexity.

The most advanced microfluidic systems for kidneys have modeled the glomerular filtration barrier (64). One notable model was created by building a three-layered glomerulus-on-a-chip (GOAC) that included a porous polycarbonate membrane coated with laminin, collagen IV, entactin, and heparin sulfate proteoglycan. The upper and lower layers of the chip contained cell culture chambers that were connected to inflow and outflow channels. Each channel demonstrated the ability to specifically mimic the afferent and efferent arterioles, respectively (64). In another model, Petrosyan and colleagues co-cultured podocytes and glomerular endothelial cells in a three-lane GOAC without the use of an artificial membrane. This Organoplate^TM^ system successfully filtered inulin and retained albumin, therefore resembling the function of the human glomerular basement membrane. The GOAC also responded to chemical injury like native glomeruli. These researchers established that the Organoplate^TM^ is useful for studying three-dimensional changes in cell morphology, abnormalities in cellular function, and crosstalk among these cells (63).

Organ-on-chip technology has many useful applications including advanced drug screening and personalized precision medicine. Within microfluidic systems, it is possible to create two-dimensional gradients of drugs to find the most effective concentration for a specific target. Organ-on-chip uses a small quantity of cells and tissues from patients, making the process easy to start because it does not require many resources. Microfluidic chips are also so small that they can be used at point of care locations. Although the technology is relatively low-cost and easy to operate, organ-on-chip systems can vary significantly between manufacturing batches (65). Overall, the low cost, high performance, and fast reaction time makes these chips convenient tools for research (66).

## Conclusion

While the kidney is an incredibly complex organ, recent advances in molecular biology, materials science, and 3D printing raise the possibility of engineering a kidney in the future. Organoids are useful yet incomplete models for a human kidney. Although they are referred to as “miniature organs,” these organoids fall short in their ability to represent the kidney due to their immaturity. Despite their limitations, kidney organoids are still very useful for high-throughput drug-screening and investigating the mechanism(s) mediating disease progression. The availability of commercial kits has also increased access to the technique of organoid differentiation, opening the doors for further innovation.

As previously stated, the use of biological and non-biological scaffolds offers solutions for some of the limitations of kidney organoid systems. 3D bioprinting technology has improved enough in recent years to offer incredibly high resolution, making it possible to print scaffolds for capillaries in which iPSCs can be implanted and propagated. Perfusion decellularization techniques have also provided useful biological scaffolds, as the ECM and its components support cell growth within the decellularized organ. Decellularization can even be used to isolate the necessary ECM components with which one can formulate tissue-specific bioinks for 3D bioprinting. Kidney-on-chip technology improves each of these techniques further by modeling the fluid dynamics in the kidney, especially within efferent and afferent arterioles. Although their sensitivity needs to be improved, glomerulus-on-chip models are convenient tools for research. Thanks to these recent technological advancements, methods for engineering kidneys to investigate disease progression and test novel therapeutics will only improve in the near future.

## Author Contributions

NCP reviewed the literature, designed, and drafted the manuscript. SKM was involved in the design and revision of the manuscript. RB and SKM intellectually contributed to the review and edited the manuscript. All authors contributed to the article and approved the submitted version.

## Funding

This work was supported by funds from the National Institute of Diabetes and Digestive and Kidney Diseases (DK112984, DK121846) and Veterans Affairs (1I01BX003698, 1I01BX005300) to SKM.

## Conflict of Interest

The authors declare that the research was conducted in the absence of any commercial or financial relationships that could be construed as a potential conflict of interest.

## Publisher's Note

All claims expressed in this article are solely those of the authors and do not necessarily represent those of their affiliated organizations, or those of the publisher, the editors and the reviewers. Any product that may be evaluated in this article, or claim that may be made by its manufacturer, is not guaranteed or endorsed by the publisher.
